# Supplementary material for: N-Palmitoyl Serinol Stimulates Ceramide Production through a CB1-Dependent Mechanism in In Vitro Model of Skin Inflammation
Source: Int J Mol Sci. 2021 Aug 2;22(15):8302. doi: 10.3390/ijms22158302 (PMC8348051; doi:10.3390/ijms22158302)
Supplement: Supplementary file 1 [file ijms-22-08302-s001.zip › ijms-1313376-supplementary.pdf]

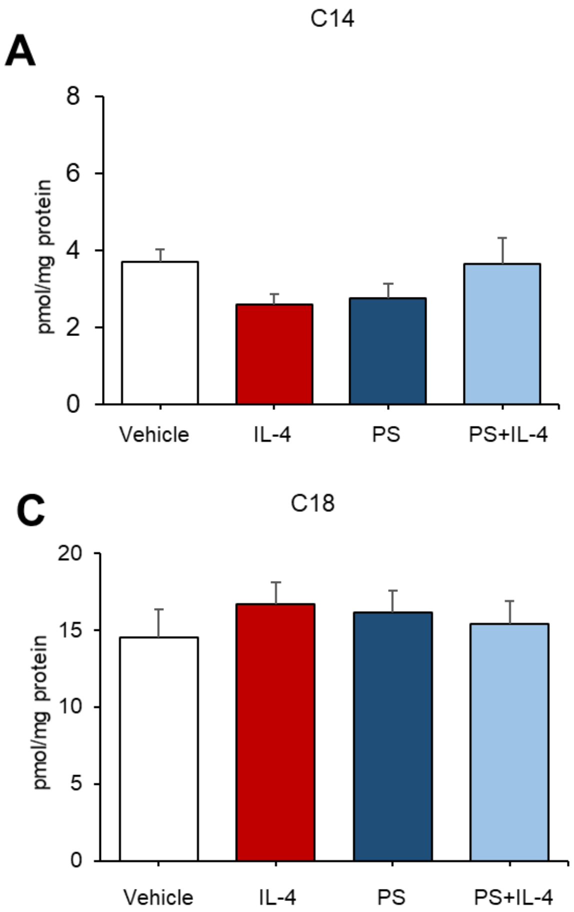

Figure S1. Changes in ceramides containing short-chain FAs (≤20) following IL-4 and/or PS treatments. HaCaT KC were pretreated with IL-4 (50 ng/mL) for 20 h, followed by incubation with exogenous PS (25 μM) for 4 h. Ceramides with different carbons in length (C16-C20, A-D, respectively) were assessed by LC-MS/MS system.
